# Analysis of rapidly synthesized guest-filled porous complexes with synchrotron radiation: practical guidelines for the crystalline sponge method

**DOI:** 10.1107/S2053273314019573

**Published:** 2015-01-01

**Authors:** Timothy R. Ramadhar, Shao-Liang Zheng, Yu-Sheng Chen, Jon Clardy

**Affiliations:** a Department of Biological Chemistry and Molecular Pharmacology, Harvard Medical School, 240 Longwood Avenue, Boston, Massachusetts, 02115, USA; b Department of Chemistry and Chemical Biology, Harvard University, 12 Oxford Street, Cambridge, Massachusetts, 02138, USA; c ChemMatCARS, Center for Advanced Radiation Sources, The University of Chicago c/o Advanced Photon Source, Argonne National Laboratory, 9700 South Cass Avenue, Argonne, Illinois, 60439, USA

**Keywords:** X-ray crystallography, crystalline sponge method, metal–organic framework, single-crystal-to-single-crystal transformation, synchrotron radiation

## Abstract

A detailed set of synthetic and crystallographic guidelines for the crystalline sponge method based upon the analysis of expediently synthesized crystal sponges using third-generation synchrotron radiation are reported. The procedure for the synthesis of the zinc-based metal–organic framework used in initial crystal sponge reports has been modified to yield competent crystals in 3 days instead of 2 weeks. These crystal sponges were tested on some small molecules, with two being unexpectedly difficult cases for analysis with in-house diffractometers in regard to data quality and proper space-group determination. These issues were easily resolved by the use of synchrotron radiation using data-collection times of less than an hour. One of these guests induced a single-crystal-to-single-crystal transformation to create a larger unit cell with over 500 non-H atoms in the asymmetric unit. This led to a non-trivial refinement scenario that afforded the best Flack *x* absolute stereochemical determination parameter to date for these systems. The structures did not require the use of *PLATON/SQUEEZE* or other solvent-masking programs, and are the highest-quality crystalline sponge systems reported to date where the results are strongly supported by the data. A set of guidelines for the entire crystallographic process were developed through these studies. In particular, the refinement guidelines include strategies to refine the host framework, locate guests and determine occupancies, discussion of the proper use of geometric and anisotropic displacement parameter restraints and constraints, and whether to perform solvent squeezing/masking. The single-crystal-to-single-crystal transformation process for the crystal sponges is also discussed. The presented general guidelines will be invaluable for researchers interested in using the crystalline sponge method at in-house diffraction or synchrotron facilities, will facilitate the collection and analysis of reliable high-quality data, and will allow construction of chemically and physically sensible models for guest structural determination.

## Abstract 2

This report describes complete practical guidelines and insights for the crystalline sponge method, which have been derived through the first use of synchrotron radiation on these systems, and includes a procedure for faster synthesis of the sponges. These guidelines will be applicable to crystal sponge data collected at synchrotrons or in-house facilities, and will allow researchers to obtain reliable high-quality data and construct chemically and physically sensible models for guest structural determination.

## 1. Introduction   

Structural elucidation is an indispensable component of chemical research, and new methods to provide rapid and accurate identification of molecular structure are constantly under development. Techniques such as nuclear magnetic resonance spectroscopy, mass spectrometry and infrared spectroscopy provide essential information regarding molecular formulas, functional groups, bond connectivities, stereochemistry and conformation. However, if one were able to grow a crystal of a target compound, then modern-day single-crystal X-ray diffraction would be the most powerful method available for obtaining all of the aforementioned information, including absolute stereochemistry, in one experiment in as little as a few hours. However, as the name implies, the limitation of X-ray crystallography is that it requires a crystal for analysis. Compounds that are liquids or amorphous solids are not amenable to this type of analysis, which excludes a very significant portion of molecular structures.

A landmark procedure for the structural determination of non-crystalline compounds using single-crystal X-ray diffraction was recently reported by Fujita and co-workers and is termed the ‘crystalline sponge method’ (Inokuma *et al.*, 2013*a* ▶, 2014 ▶). Their procedure involves a thermodynamically favorable exchange of solvent with microgram to nanogram quantities of a target guest molecule within a crystalline metal–organic framework (MOF). The MOF with encapsulated guest can then be subjected to single-crystal X-ray diffraction, which should afford the structure of the host and guest. The crystal sponge employed most frequently in their studies is {[(ZnI_2_)_3_(tris(4-pyridyl)-1,3,5-triazene)_2_]·*x*(solvent)}_*n*_ (Birad­ha & Fujita, 2002 ▶). Furthermore, the presence of heavy atoms within the host framework, coupled with the guest molecule’s induction of a single-crystal-to-single-crystal trans­formation, allows the absolute configuration of molecules that do not contain heavy atoms to be unambiguously determined using ubiquitous Mo *K*α irradiation sources on in-house diffractometers. As an aside, the study of guests enclatherated in host networks is not novel and was performed over two decades ago where guest molecules were co-crystallized within porphyrin networks, and it is of interest to note that the authors explicitly call these systems ‘sponges’ within the article’s title (Byrn *et al.*, 1993 ▶). Nonetheless, the report by Fujita and co-workers attracted significant attention from the scientific community due to its potential to revolutionize the practice of structural elucidation (Stallforth & Clardy, 2013 ▶; Callaway, 2013 ▶; Halford, 2013 ▶). However, as time progressed, it became evident that data quality was an issue for this method and eventually a correction for the original report was published that pertained to the incorrect stereochemical determination of miyakosyne A (Inokuma *et al.*, 2013*b* ▶). Nevertheless, Buchwald and co-workers recently described the application of the crystalline sponge method, in conjunction with other analytical methods, for the structural determination of an electrophilic trifluoromethylthiolation reagent that was found to be a thioperoxide (Vinogradova *et al.*, 2014 ▶). Fujita and co-workers subsequently described a variant of the solid-state dilution method (Coppens *et al.*, 2006 ▶, 2008 ▶; Coppens & Zheng, 2011 ▶) to study the mechanism of Pd-catalyzed aromatic bromination using a substrate that was enclatherated within the sponge during crystal growth (Ikemoto *et al.*, 2014 ▶), in a manner resembling the X-ray crystallographic studies of the ultraviolet-light-induced formation and proposed observation of 1,3-dimethylcyclo­butadiene in a guanidinium sulfonate–calixarene crystalline network with reaction intermediate monitoring (Legrand *et al.*, 2010*a* ▶,*b* ▶; Alabugin *et al.*, 2010 ▶; Scheschkewitz, 2010 ▶). While the method certainly promises more applications in the near future, the issue of data quality persists as the current method does not yield results that meet accepted crystallographic standards (Extance, 2014 ▶).

**Figure 1:**
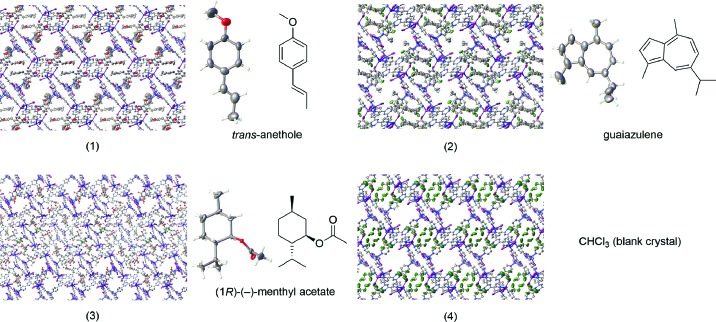
Crystal sponge complexes studied through synchrotron radiation. These structures are available as supporting information and have also been deposited in the Cambridge Structural Database (CSD) [(1) CCDC 1007931, (2) CCDC 1007929, (3) CCDC 1007930, (4) CCDC 1007932].

We were intrigued by the possibility of using the crystalline sponge method for the study of natural products and other biologically active molecules. Preliminary studies for this method with chemically simpler guests such as *trans*-anethole, guaiazulene (Inokuma *et al.*, 2013*a* ▶, 2014 ▶) and (1*R*)-(−)-menthyl acetate (Fig. 1 ▶)^1^ led us to encounter general operational and crystallographic issues that were unexpected for these systems. Further studies with high-flux third-generation synchrotron radiation allowed us to explore the entire process of the crystalline sponge method in detail. Herein we present the results of those initial studies, which include a much faster synthesis of viable crystal sponges and a thorough set of crystallographic guidelines and procedures from crystal selection, data collection, indexing, integration, scaling, space-group determination, phasing and structure refinement. We describe the general challenges associated with the crystal sponge method and how problems arising from the analysis of in-house data for (1) and (3) were overcome by using synchrotron radiation. Chemical crystallographic analysis of the reported crystal sponge data allowed for unambiguous location of guest molecules for structural identification. The presented information will be of value for those interested in performing the crystalline sponge method using either in-house diffractometers or synchrotron radiation, and the presented guidelines will allow researchers to consistently obtain high-quality data in order to build chemically and physically sensible models that can be used for reliable guest structure determination.

## 2. Results and discussion   

### 2.1. Crystal growth, guest inclusion and crystal selection   

**Figure 2:**
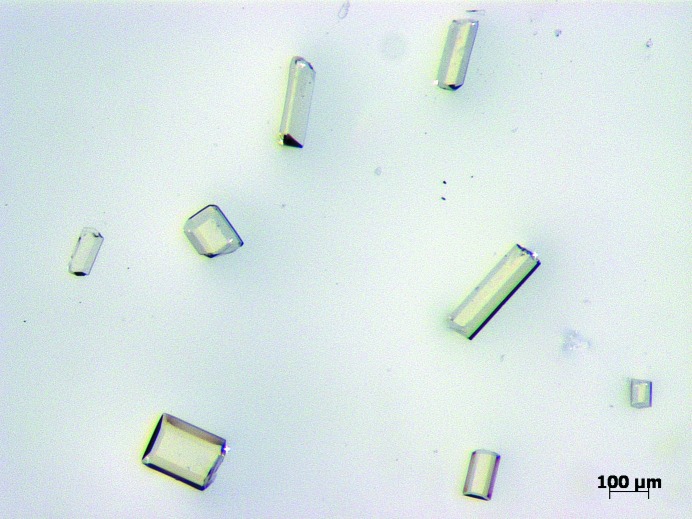
Ideal crystal sponges for guest inclusion. These crystals were preselected from a batch containing crystals with other morphologies (*e.g.*, flat sheets and twinned microcrystals) for the purposes of this photograph.

We were interested in developing a method to generate the ZnMOF crystal sponges with greater operational simplicity and speed. We found that growth conditions similar to those reported by Fujita and co-workers (Inokuma *et al.*, 2013*a* ▶, 2014 ▶) where CHCl_3_ was used instead of nitrobenzene generated suitable crystals of {[(ZnI_2_)_3_(tris(4-pyridyl)-1,3,5-triazene)_2_]·*x*(CHCl_3_)}_*n*_ for guest inclusion within 3 d (Fig. 2 ▶). These crystals did not require solvent exchange, were ready for immediate use and could be stored at 253 K. Three types of crystals were obtained from this procedure: narrow/wide prisms, thin sheets, twinned microcrystals. The narrow/wide prisms were the only crystals suitable for analysis.

This synthetic procedure is much faster than the original method (Inokuma *et al.*, 2014 ▶), where crystals were grown in nitrobenzene/MeOH over 7 d and required exchange of nitrobenzene with cyclohexane *via* heating at 323 K for an additional 7 d. The new procedure is also milder since it does not expose the crystals to heat for long periods of time, thereby reducing the risk of introducing crystal imperfections (*e.g.*, increase in mosaicity). The crystals were prepared using a minimum number of solvents as they are exposed only to CHCl_3_/MeOH during synthesis and are washed and stored in CHCl_3_. The previous procedure for ZnMOF synthesis required the use of nitrobenzene/MeOH and cyclohexane (Inokuma *et al.*, 2013*a* ▶, 2014 ▶). A concern about the use of nitrobenzene and cyclohexane is that it may complicate structure refinement, and it is possible for nitrobenzene to remain within the ZnMOF after a solvent-exchange procedure (Vinogradova *et al.*, 2014 ▶). If the target guest molecule contains cyclohexyl or aromatic rings, then it may be difficult to distinguish the guest from residual solvent if the site occupancy is low and/or the data quality is poor. Careless use of hard crystallographic restraints could lead one to incorrectly model the desired guest molecule using residual solvent electron density. Furthermore, if the guest and residual solvent interact with the host in a similar manner (likely to be problematic if they both contain aryl or cyclohexyl rings), then the likelihood of occupational disorder increases and structure refinement may become more challenging. In contrast, any CHCl_3_ that might remain within the ZnMOF after guest inclusion can be easily observed due to the longer C—Cl bond length (∼1.7 Å) and larger Cl electron density. Though the greater electron density for CHCl_3_ (58 electrons) *versus* the more diffuse electron density for cyclohexane (48 electrons) and nitrobenzene (64 electrons) will exert a slightly larger influence on the structure factors relative to incorporated guest, it is outweighed by the aforementioned benefits of CHCl_3_ usage.

For the guest inclusion experiment, the crystals were submerged in either neat *trans*-anethole, guaiazulene or (1*R*)-(−)-menthyl acetate at ambient temperature for 2 d in a small glass vial. (*Note*: the melting point of guaiazulene is 300–302 K. It can be liquefied for use and will not immediately revert to a solid form; should it solidify during inclusion, then it can be re-liquefied by gentle warming.) We generally recommend that crystals are soaked in neat target guest molecules for the inclusion experiment, which was also done by Buchwald and co-workers for a thioperoxide guest (Vinogradova *et al.*, 2014 ▶). This is strongly advised in order to ensure that a sufficient quantity of guest is included within the crystal, because it could be exceedingly difficult to locate and model the guest with reasonable confidence if the site occupancy or data quality is low. While part of the allure for the original crystal sponge report was the study of guests at microgram and nanogram quantities (Inokuma *et al.*, 2013*a* ▶), the other significant aspect of the method is the ability to elucidate the structures of compounds that are difficult or impossible to crystallize – such as the analysis of liquids. Though some guests may be amenable to analysis in limited quantities, we suggest the use of neat guest in order to maximize the *generality* of the method. Future experiments will aim to reduce the guest concentration. The crystals typically withstood soaking in neat liquid guests, with *trans*-anethole being the harshest on crystal quality. Additionally, the risk of introducing crystal imperfections during the inclusion experiment through heating was avoided, though it is possible to warm these crystals to 323 K in an analogous manner to the described protocol by Fujita and co-workers, if necessary (Inokuma *et al.*, 2013*a* ▶, 2014 ▶).

Multiple crystals were concomitantly soaked in the same vial for all guest inclusion experiments. The reasons for this are twofold. First, if the guest has a propensity to degrade crystallinity, then soaking more crystals at once increases the probability of finding a high-quality crystal. Second, and most importantly, the best method to select for high-quality crystals is by viewing the reflections on the reciprocal lattice, which can only be observed from performing initial diffraction experiments. Only from viewing the reciprocal lattice can imperfections arising from minor cracking and twinning be observed. While the crystal-picking method by Fujita and co-workers can be performed, where a crystal is selected by visual inspection prior to the soaking experiment (Inokuma *et al.*, 2014 ▶), it is highly recommended that multiple soaking experiments are set up in parallel in order to increase the probability of finding the best quality crystal as judged by reciprocal-lattice evaluation. Therefore we recommend that multiple crystals are concomitantly soaked in one vial, which increases the general ease and efficiency of setup for guest inclusion, crystal picking and mounting. After guest inclusion experiments, crystals were manipulated in NVH immersion oil for selection and mounting. The use of a polarizing light filter can aid in visualizing cracking; however, the best gauge for quality is performing initial diffraction studies. It is important to note that the crystals should not be shipped to synchrotron sources in the immersion oil. We have observed problems with performing diffraction studies on these crystals, which may possibly arise from diffusion of the guest within the sponge into the immersion oil.

### 2.2. Data collection, processing and structure solution   

Analysis of the crystal sponges greatly benefitted from the familiar advantages of synchrotron use. Synchrotron data were collected with flash cooling using a 100 K liquid-nitrogen cryostream, where the low collection temperature should reduce disorder while protecting the crystal against radiation damage. The ZnMOF crystals survived high-flux synchrotron radiation for the collection of full data sets. A much shorter exposure time was used at the synchrotron compared to in-house sources. For diffractometers with sealed-tube Mo *K*α anode sources, an exposure of 2 min per frame for the crystal sponges was required in order to attempt to collect data at sufficient resolution and required multiple days for collection time. However, the data were significantly weaker in the high-resolution shells. Exposure times at the synchrotron were minimized to 0.3 s per frame and allowed for the collection of full data sets consisting of multiple runs with good resolution in less than 1 h. Depending on the size and quality of the crystal, it may be occasionally necessary to use attenuation filters in order to prevent consistent oversaturation of the low-angle reflections. Problematic overloaded reflections can also be omitted during refinement for typical chemical crystallography studies. Data were collected using a wavelength of ∼0.41 Å on account of much lower absorption by the heavy atoms in the host. This wavelength allows for better absorption correction and mitigates the use of extinction or diffuse solvent corrections. Intriguingly, collection of the data with high-flux radiation at this shorter wavelength led to the observation of green light emanating from the crystal sponges due to X-ray fluorescence, which was not observed with in-house diffractometers.

Another advantage of using the synchrotron for these systems occurs when the unit-cell dimensions change to incorporate a long axis *via* a single-crystal-to-single-crystal transformation process. This occurred for the inclusion of (1*R*)-(−)-menthyl acetate. While (4) exhibited a monoclinic *C*2/*c* space group with a *c*-axis length of 31.081 (3) Å, the inclusion of (1*R*)-(−)-menthyl acetate to afford (3) changed the space group to monoclinic *P*2_1_ with the *c*-axis length expanded to 66.990 (6) Å. Long unit-cell axes correlate to small reflection distances in reciprocal space. If the detector distance is small (*e.g.*, the typical 5 cm distance used on in-house diffractometers), then the reflections will overlap. These overlapped reflections could cause indexing programs to select the incorrect Bravais lattice and unit-cell dimensions or fail, and space-group determination can be hampered through incorrect presence/lack of systematically absent reflections. Even if the data are solved in the correct space group and unit cell, the data quality will be poor. While the typical solution to this problem is to increase the detector distance from the crystal, this is not suitable for analysis of crystal sponge systems on in-house diffractometers with a sealed-tube anode Mo *K*α source, since the reflection intensities would be extremely weak or absent, especially in the mid- to high-resolution shells. Measuring reflections in-house using 2 min per frame exposures with a 5 cm detector distance is at the limit of what can be done on these diffractometers; increasing the detector distance will not provide usable results. However, high-flux synchrotron radiation will allow for successful data collection of these systems with larger detector distances. Using synchrotron radiation, a 0.3 s exposure with a 10 cm detector distance allowed the collection of high-resolution data for (3) with sufficient intensity, where the 0.869–0.839 Å shell exhibited a percentage of merged reflections with intensities < 2σ(*I*) at 77.1%. The in-house data were significantly worse and overlaying the refinement results with the reflections collected in-house and processed in space group *P*2_1_ led to a larger *R*_1_ value and made >350 non-H atoms become non-positive definite (NPD).

As mentioned previously, the best manner to determine the quality of the crystal is to view the reciprocal lattice after multiple frames are collected. Data collection should be aborted if the crystal exhibits significant cracking, non-merohedral twinning, or is suspected of pseudo-merohedral twinning. The crystal sponge systems are not trivial to process or refine; therefore, additional complexities arising from crystal deficiencies should be avoided. It is extremely difficult to determine whether guest incorporation has occurred after performing a set of matrix scans. The most definitive sign that inclusion has occurred for these systems is if indexing reveals that the crystal has significantly different unit-cell parameters than (4), which was observed for (3). No difference in the Bravais lattice or minimal changes in the unit-cell angles or axis lengths are problematic and will require full data collection, processing and refinement to determine whether inclusion has occurred. A significant advantage of using a synchrotron-radiation source is that the data-collection time for the crystal sponges is reduced from days to less than 1 h, which makes screening faster, maximizes beam time and reduces financial cost.

The process of indexing, integration, multi-scan absorption correction (Bruker, 2012 ▶) and space-group determination was routine for all reported crystal sponge synchrotron data. However, processing of the analogous in-house data for (1) and (3) was much less straightforward due to systematically weaker reflection intensities. In the case of the in-house data for (1), the indexing program selected a triclinic *P* Bravais lattice, smaller unit cell, and the space group was assigned as *P* 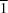. However, the indexing program was able to determine a monoclinic *C* Bravais lattice with a larger unit cell for the analogous synchrotron data. The space group was assigned as higher symmetry *C*2/*c*. Processing of the in-house data for (3) was problematic, which was likely complicated by the long *c* axis, and only triclinic *P*1 appeared to be the most reasonable space-group choice with the *R*_sym_ for *P*2_1_ being 0.167 even when the data were indexed, integrated and scaled assuming a *P*2_1_ space group. However, the processing of the analogous synchrotron data was straightforward and the space group was unambiguously assigned as chiral *P*2_1_ with a lower *R*_sym_ value of 0.051. Care must be taken if in-house data are used given the difficulty that can be experienced for data processing. If indexing or space-group determination is problematic, it may be worthwhile to process the data assuming a triclinic *P* Bravais lattice and then do a careful search for valid unit cells with higher metric symmetry followed by thorough examination of systematic absences to choose the correct space group, which will necessitate reprocessing of the data. This process is further complicated if the crystal contains lattice imperfections.

For the structure-solution process, the use of intrinsic phasing afforded great starting models on which to start the refinement process. Intrinsic phasing (*SHELXT*, or Bruker *SHELXTL XT*) uses an admixture of direct, Patterson and dual-space methods (Sheldrick, 2008 ▶; Ruf & Noll, 2014 ▶). Intrinsic phasing first expands the data set to triclinic *P*1, which plays to the power of direct methods; however, it starts the structure-solution process from a Patterson superposition minimum function. The phases are then used to determine the space group in order to improve the initial electron-density map, followed by subsequent refinement using dual-space recycling with random omit maps. The free-lunch algorithm is also used to improve the electron-density map, where the phases of reflections that have not been measured are extrapolated to a given resolution (Caliandro *et al.*, 2005 ▶, 2007 ▶). Intrinsic phasing will assign atom types to the final map on positions of greatest electron density. For all presented ZnMOF–guest complexes, intrinsic phasing was able to locate and assign atom types for the host framework including portions or complete guest molecules for (1) and (3). Intrinsic phasing was also observed to be very tolerant of the input molecular formula given for structure solution.

While intrinsic phasing can be a powerful method for solving crystal sponge synchrotron data, it is also worthwhile to investigate a few alternative phasing methods; thus, a cursory study of other phasing methods was performed using the default program settings. Fujita and co-workers recently reported the use of *SUPERFLIP* (Palatinus & Chapuis, 2007 ▶), which is based upon charge flipping, to solve their ZnMOF structures. A combination of *SUPERFLIP* and *Electron Density Map Analysis* (*EDMA*) (Palatinus *et al.*, 2012 ▶) as implemented in the *WinGX* software suite (Farrugia, 2012 ▶) was tested on the synchrotron data to further assess its applicability to these systems. While it was observed that *SUPERFLIP* + *EDMA* with the default program settings did not produce starting models that were of the same quality as those constructed through intrinsic phasing (disjointed portions of host framework were located and complete guest fragments were not located), it did afford usable starting models on which to continue successful refinement. The quality difference in the *SUPERFLIP* + *EDMA* model may be attributable to the fact that the presence of heavy atoms can cause the charge-flipping algorithm to lose light atoms in the noise (Oszlányi & Sütő, 2008 ▶). Reasonable starting models can be generated through the use of the *SirWare* programs [*e.g.*, *SIR-92* (Altomare *et al.*, 1994 ▶), *SIR-2008* (Burla *et al.*, 2007 ▶) or *SIR-2011* (Camalli *et al.*, 2012 ▶)], which are based on direct methods and use a modular approach involving structure invariant/semi-invariant estimation and phasing. The success of *SirWare* methods with the default program settings in building a good starting model is highly dependent on the resolution cutoff, where a 0.1 Å differential (*e.g.*, 0.9 Å *versus* 1.0 Å) can play a marked difference in obtaining a good solution. Dual-space methods (Bruker *SHELXTL XM*) (Sheldrick, 2008 ▶) were also tested and could generate a reasonable starting model that contains a fragmented host. Furthermore, the ability of *SirWare* and dual-space methods to construct decent starting models for these systems appears to exhibit greater dependence on input molecular formula, whereas intrinsic phasing is more tolerant. Again, it is important to note that the default program settings were used and it may be possible to fine tune these phasing methods *via* careful parameter optimization to further improve the starting model.

### 2.3. Refinement strategy   

#### 2.3.1. Refining the host framework   

Upon obtaining a viable starting model, focus should first be placed on constructing the host framework through least-squares refinement. This should be a priority because once the host framework is sufficiently modeled, then residual electron density for the guest molecules will become much more obvious. As mentioned previously, intrinsic phasing can generate a good starting geometry for the host framework if high-quality data are used, though correction of the atom types and assignment of missed electron density will be required. If other methods are used, the host framework may be significantly fragmented since the unit cell contains multiple ZnI_2_/ligands. These fragments should be carefully reassembled to form a contiguous host moiety in order to facilitate refinement and to depict a chemically sensible model. If unit-cell centering operations are performed on a model derived through intrinsic phasing to place the center of gravity within or near the bounds of the unit cell, then some minor fragmenting of the host pyridine and triazine rings may occur due to its polymeric nature and can be easily recovered.

A departure from previously reported ZnMOF crystal-sponge–guest complexes involves the treatment of residual electron density near the Zn and I atoms in the host. For the ZnMOF crystal sponge data obtained from the synchrotron, this residual electron density was treated as positional disorder of Zn and I. The possibility that this residual density arose from poor absorption correction is low since it was anisotropically modeled in a stable fashion. Furthermore, problems arising from the absorption of X-ray radiation from Zn and I are largely mitigated when using short-wavelength synchrotron radiation. The occurrence of positional disorder for these atoms is chemically sensible. It is expected that guest and solvent molecules associate with the host through van der Waals (vdW) interactions. These interactions will likely induce a geometrical distortion of the host framework. However, the spatial average of the unit cell is complicated by partial occupancies of the guest and solvent; therefore positional disorder of the Zn and I atoms will arise. The Zn and I atoms are more distortable due to the longer Zn—I (∼2.5 Å) and Zn—N (∼2.0 Å) bonds, and therefore this effect is more readily observed for these atoms *versus* the remainder of the host.

The positional disorder for Zn and I can be free refined as simple two-component disorder as performed for (1), (2) and (4), or as three- and four-component disorder as performed for (3) where the occupancy of all components sums to unity. Local exact anisotropic displacement parameter (ADP) constraints, such as the EADP instruction in *SHELXL*, were used to model pairs or sets of equivalent disordered atoms (Zheng *et al.*, 2007 ▶). It was occasionally necessary to use soft local distance restraints based upon sets of equivalent bonds, such as the SADI directive in *SHELXL*, on the disordered Zn—I and Zn—N bonds, and an exact positional constraint (*e.g.*, *SHELXL* EXYZ) on a Zn atom in (1) was required. The disorder was isotropically modeled and then anisotropically refined with multiple least-square cycles in order to determine its occupational stability. For (3), some Zn/I disorder that appeared reasonable in an isotropic model proved to be unstable once multiple anisotropic refinement cycles were performed (the occupancy changes to 100%/0%). Modeling the positional disorder on Zn and I reduced the *R*_1_ and *wR*_2_ refinement statistics. The biggest difference was observed for (1), where the *R*_1_ was reduced by 3.6% and the *wR*_2_ was lowered by approximately 8.4%. Furthermore, the Flack *x* parameter (Parsons *et al.*, 2013 ▶) for (3) increased by a factor of approximately 2.5 if Zn/I disorder was not modeled.

#### 2.3.2. Locating guests   

Once the host framework is anisotropically refined, focus should then be placed on locating guest and solvent molecules. Structure-solution methods may be able to locate some guest molecules, albeit with some incorrect atom types. These guests are typically the most ordered within the unit cell. For other guest and solvent molecules that have not been modeled through intrinsic phasing or other structure-solution methods, it is necessary to locate them through careful search of the residual electron density. It is helpful to increase the amount of visible residual electron-density peaks (Q-peaks) to 90 or greater, and 500 for the case of (3) due to its size. Structure-solution methods may generate erroneous atoms that do not make chemical sense, which was observed in all cases. For these situations, it is best to delete these atoms, perform least-squares refinement cycles, and determine if the recovered electron density has significance. It is extremely helpful to use symmetry-generation tools to perform centering on cell operations, growing and re­assembling disjointed fragments. For all reported host–guest complexes, these symmetry-generation tools relocated Q-peaks and made guest-atom location more straightforward. If part of a guest can be modeled, though some atoms are missing, it is worthwhile to manually reduce the occupancy of the guest to 50% or lower and determine whether the residual electron density for the missing atoms appears. However, once all atoms have been located, it will be necessary to free refine the occupancy. These strategies were successfully applied to locate (within the asymmetric unit) three guests in (1), two guests and two CHCl_3_ in (2), 14 guests and one CHCl_3_ in (3), and three CHCl_3_ guests in (4). It is interesting to note that the conformation of the *trans*-anethole guests in (1) differs from that of the same guest within the previously reported porphyrin sponges obtained through co-crystallization (CSD reference code: HALWIA) (Byrn *et al.*, 1993 ▶; Allen, 2002 ▶).

Atom types need to be assigned correctly, and both isotropic ellipsoid size and bond distances can be a helpful indicator of correct/incorrect assignment. This is easiest for highly occupied and highly ordered guests. However, since factors involving quality, occupancy and disorder (including errors arising from Hirshfeld rigid-bond tests) can make atom-type assignment difficult, it is strongly recommended that a molecular formula obtained from elemental analysis (EA) or high-resolution mass spectrometry (HRMS) be used as guidance for an unknown structure. Unlike some previously reported ZnMOF–guest structures (Inokuma *et al.*, 2013*a* ▶; Ikemoto *et al.*, 2014 ▶), *all* non-hydrogen atoms were anisotropically modeled in the current crystal sponge systems. Anisotropic modeling depicts a more reasonable chemical model of the system in regard to electron distribution, and it can potentially reduce the *R*_1_ and *wR*_2_ refinement statistics provided that high-resolution data are used. Careful application of ADP restraints can be used in the event where problematic anisotropic thermal ellipsoids that have severe prolate/oblate distortions arise due to disorder or data-quality issues (see below). There is no strong justification for leaving any isotropically modeled fragments within the general realm of chemical crystallography when data of reasonable resolution are collected.

**Figure 3:**
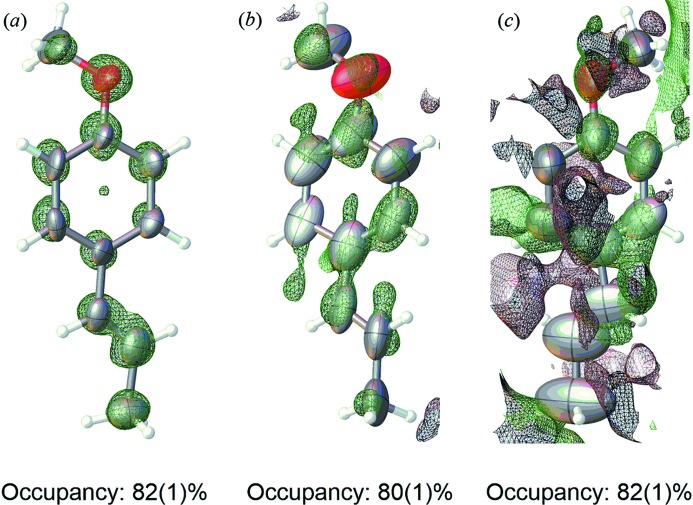
Comparison of three-dimensional difference (*F*_o_ − *F*_c_) maps for three *trans*-anethole guests with occupancies and varying map intensities (green: positive density, red: negative density, *Olex2* (Dolomanov *et al.*, 2009 ▶); map levels: (*a*) −4.02, (*b*) −3.12, (*c*) −1.86. The final *trans*-anethole structures have been manually overlaid on the electron-density maps using the *Olex2* overlay tool.

A check of guest electron density should be performed by analyzing Fourier electron-density maps (Fig. 3 ▶). While the host is clearly visible from *F*_o_ maps, the electron density for guest and solvent is generally weaker. Difference maps (*F*_o_ − *F*_c_) are particularly helpful in determining the presence of guest electron density. While the three *trans*-anethole guests share very similar high occupancies, the difference maps clearly show that this does not necessarily translate to well defined electron density. Additionally, the density may not be visible if geometric restraints were required to shift the atoms into chemically reasonable positions such as the alkenyl carbon atoms for the most disordered guest (Fig. 3 ▶ *c*). However, the difference map indicated no significant negative regions of electron density, indicating that the placement of those atoms is justified despite the electron density being minimal and flat. Furthermore since the crystals were only exposed to CHCl_3_/MeOH during synthesis and *trans*-anethole for guest inclusion, these atoms can logically be assigned by virtue of the observed density on the other atoms and by their observable Q-peaks during isotropic refinement. However, this line of reasoning is more difficult to justify if the crystal sponge contained residual solvent bearing an aromatic ring, or if a guest molecule and residual solvent share similar moieties such as a cyclohexyl ring. Thus it is important that any residual solvent is significantly different than the desired guest molecule, otherwise there is a risk of assigning residual solvent electron density as the target molecule. This logic also encourages the use of neat guest for inclusion in order to maximize occupancy, thereby facilitating the assignment of guest electron density, especially if disorder is a significant factor.

In cases where a portion of the guest is missing (*i.e.*, no Q-peaks are visible), it is possible that the corresponding atoms are too disordered to locate or a guest molecule may not be present (noise may have been mistaken for guest electron density). If modeling of the suspected guest cannot be completed, then it is advisable that the incomplete molecule be removed. It may not be worthwhile (or accurate) to continue modeling the problematic guest. Any decision to continue its modeling must be justified through the Fourier electron-density maps. Overall, it is better to err on the side of caution for the analysis of the crystal sponge systems than to make structural claims that cannot be adequately supported by experimental data, otherwise incorrect conclusions may be drawn.

#### 2.3.3. Geometry restraints   

Depending on data quality and both the occupancy and disorder of guest and residual solvent molecules, it may be necessary to apply geometric restraints to generate a chemically reasonable model. Nonetheless, there were one or more target molecules in (1), (2) and (3) that did not require the use of geometric restraints. In these cases, it is best to use the ideal guest molecule in order to assist in the modeling of the distorted molecules through soft restraints based on equivalent sets, such as the SADI and SAME commands in *SHELXL*, which make 1,2 and 1,3 bond distances equivalent within a specified standard deviation, thus not requiring the introduction of explicit bond distances or angles. A prior literature recommendation for the use of harder bond and geometry restraints that introduce explicit assumptions based upon theoretical values, such as the DFIX and FLAT directives in *SHELXL*, for the crystal sponges exists (Inokuma *et al.*, 2014 ▶), and hard angular restraints (*e.g.*, *SHELXL* DANG) might also be grouped in this category. The risk of using these hard restraints is that the geometrical features of the molecules, particularly the target guest molecules – which are supposed to be based purely on experiment – are lost due to the incorporation of theoretical values, especially when these hard restraints are used in excess. Softer restraints based upon equivalent sets, in contrast, allow one to use intuitive chemical knowledge that related bond distances in the guest are supposed to be similar. In the case where only one guest or residual solvent molecule is present and is distorted, then soft restraints can logically be used within the guest to set chemically sensible restraints between undistorted and distorted bonds/angles (*e.g.*, use soft restraints to make all C—Cl bond distances similar for distorted CHCl_3_, or take advantage of internal symmetry to set similar bond distances in guest molecules *etc*.). Therefore, hard restraints should only be applied to the crystal sponge systems under exceptional circumstances, used very sparingly with explicit justification, and *never* before attempting soft restraints. In no case for (1), (2), (3) or (4) was it necessary to use hard restraints; only the use of soft restraints was required. They were used lightly and when necessary in order to create a stable and sensible model for the distorted molecules within the crystal sponge without over-modeling and only if the residual electron density was confidently assigned as belonging to the guest.

Other geometric issues that arose involved the treatment of riding-model-added H atoms on methyl groups. Significant maximum shift/estimated standard deviation (e.s.d.) errors on these positions can occur and will be listed in a *checkCIF* validation report (http://checkcif.iucr.org) (Spek, 2009 ▶). This is especially problematic for methyl groups with significant disorder, and was observed for (2) and (3). Restraining the ADP parameters on the methyl carbon may help in eliminating these maximum shift/e.s.d. errors. However, the most effective manner in dealing with this problem, especially with lower-quality data, is to prevent the methyl H atoms from rotating in the refinement to avoid a futile fitting attempt in regions where the electron density is very weak and/or smeared. Another issue that arose for (3) was the occasional incorrect binding of disordered host Zn atoms that exhibit large angular distortion to proximal pyridine C or H atoms. These spurious bonds were explicitly removed from the connectivity lists.

Finally it is worth mentioning that despite collecting the data at a synchrotron, *checkCIF* level C alerts for both (1) and (2) and a level B alert for (3) for low C—C bond precision occurred. This is likely a reflection of both the overall data quality and partial guest occupancies/disorder within the crystal sponges. Data quality may be a larger contributing factor for (3) due to the larger detector distance employed during data collection. Since the primary use of the crystalline sponge method is to determine bond connectivities and relative/absolute stereochemistry of the guest molecules, the level B and C low C—C bond precision alerts are tolerable.

#### 2.3.4. Anisotropic displacement parameter constraints and restraints   

The nature of the data quality and guest/residual solvent molecule ordering within the crystal sponge system necessitated the use of ADP restraints. Furthermore, it is important to note that high guest occupancy does not necessarily translate to ideal ellipsoid appearance. In all reported ZnMOF–guest complexes, the guest molecules with the ellipsoids with the best size and shape forge significant vdW interactions with the host framework. Disorder is therefore problematic when the guest is weakly stabilized within the cavity. Any level of disorder will impart pathological ellipsoidal geometries (prolate or oblate spheroids), and it may be extremely impractical or unreasonable to create a whole-molecule disorder model for the guests. Therefore application of ADP restraints is certainly justified for the crystal sponge systems; however, it must be done with care.

In regard to the host framework, equivalent ADP constraints were applied for the disordered Zn and I atoms. They were applied only locally for disordered pairs or sets of the same atom type (*i.e.*, a single equivalent ADP constraint instruction did not encompass all Zn or I in the unit cell) (Zheng *et al.*, 2007 ▶). This forces the ADP parameters of the atom pairs/sets to be equivalent and is especially important for overlapping atoms since they share electron density, creating an interdependence between their occupancies and thermal factors. Constraining these pairs allows them to share the same ADPs (which makes chemical sense as they should be very similar) and removes this dependency, allowing for more accurate refinement of the site occupancy. Attempts to replace equivalent ADP constraints with restraints that impose the same *U*_*ij*_ components within an effective standard deviation, such as the SIMU command in *SHELXL*, were performed for (1), (2) and (4). While this can lower the *R*_1_ by 0.3–0.7%, the ellipsoids exhibit pathological shapes that trigger level C ADP alerts (see below) or can cause an atom to become NPD. Furthermore, the occupational uncertainty changes unfavorably by two- to fivefold. Thus equivalent ADP constraints were used for Zn/I disorder in the crystal sponge systems because while the *R*_1_ increases slightly, the occupational uncertainty values are much lower, which is more desirable from an experimental standpoint.

For pathologically shaped ellipsoids in the host and guest, standalone use and combinations of ADP restraints that impose (i) a rigid-bond restraint where ADP components in the direction of the bond are made equal within an effective standard deviation (*e.g.*, *SHELXL* DELU), (ii) the same *U*_*ij*_ components within an effective standard deviation (*e.g.*, *SHELXL* SIMU), and (iii) recently described enhanced rigid-bond restraints (*e.g.*, *SHELXL* RIGU) (Thorn *et al.*, 2012 ▶) were employed. Typically restraints of type (i) and (i) + (ii) were used on disordered atoms in the crystal sponge systems first; however, if these were insufficient, then a restraint of type (iii) replaced (i). These restraints were applied only on fragments or entire guest molecules. For the host framework, only small fragments of the pyridine and triazine host moieties required ADP restraints. Applying ADP restraints globally is best avoided since this could unnecessarily muddle the chemical meaning of anisotropic electron distribution for all non-H atoms. Furthermore, the use of equivalent ADP constraints to model different atom types in fragments or entire guest molecules that have not been split into separate disordered components is not to be performed, since atoms that should not have the same ADPs will be constrained to have the same values – the chemical meaning of anisotropic electron distribution could be entirely lost (Ikemoto *et al.*, 2014 ▶). In all four reported ZnMOF–guest complexes obtained from the synchrotron, some guests contained extreme oblate/prolate ellipsoids after starting anisotropic modeling. In these cases, a combination of ADP restraints of types (i) + (ii) and (ii) + (iii) proved to be extremely helpful, with the latter being a more powerful restraint combination. The use of ADP restraints that enforce approximate isotropic behavior on the *U*_*ij*_ components, such as the ISOR command in *SHELXL*, should be avoided if possible and used only as a last resort if a combination of ADP restraints of (ii) + (iii) cannot resolve a severely oblate/prolate ellipsoid (level A/B ADP ratio alert). Problems with NPD ellipsoids are observed if there are data-quality issues, or if the occupancy is very low. NPD atoms were observed primarily in (3) and were most prevalent in a (1*R*)-(−)-menthyl acetate molecule at 27 (1)% occupancy. The combination of ADP restraints of types (ii) + (iii) on entire molecules or fragments proved to be extremely helpful in removing NPD atoms. These ellipsoids may also originate from severe disorder, poor data integration/scaling, incorrect space-group choice or use of a poor-quality crystal. It is important to note that while the refinement was performed in *SHELXL-2014* for the current crystal data, other software packages such as *CRYSTALS* (Betteridge *et al.*, 2003 ▶) will allow for the application of similar restraints and constraints.

It is important to not over-model the structure when restraining ADPs. Restraints in the crystal sponge systems were applied until the structure did not afford *checkCIF* level A/B ADP ratio alerts. If only a few level C ADP ratio alerts exist, then the affected atoms can be restrained until no ADP ratio alerts appear, if desired. However, in the case of (3), ADP restraints were applied with 31 level C ADP ratio alerts being afforded after final refinement. In this case, it was best only to address level A/B ADP ratio alerts and ignore the level C alerts. The presence of these alerts arises from disorder and data quality. The latter is heightened for (3) since the CCD detector was moved farther away for data collection, which reduces reflection intensities especially in the high-angle shells and affects both completeness and redundancy in those shells. Trying to address these level C alerts with ADP restraints will introduce hundreds or thousands of restraints and the over-modeled system will not truly reflect the experimental data in terms of quality and substantial disorder. The *checkCIF* validation tests may also return Hirshfeld rigid-bond test errors, which also necessitates additional experimental data such as a molecular formula from HRMS or EA in order to confidently model the structure.

#### 2.3.5. Guest occupancies and whether to treat residual density in solvent-accessible voids   

Determination of guest and solvent occupancies is necessary to properly refine the model and to gain further chemical insights into the system. From analysis of the synchrotron data, the best way to determine occupancy for the crystal sponge systems was to free refine it by assigning a unique site-occupancy factor for each guest that is refined. This method establishes the approximate minimum occupancy for each free refined molecule. Intriguingly, it was observed that the occupancies for the guest molecules within (1) *increased* when *PLATON/SQUEEZE* was tested on this system. The occupancy for one guest molecule increased from 82 (1)% to 104 (1)%, while another increased from 82 (1)% to 94 (1)% and the final guest molecule increased from 80 (1)% to 88 (1)%. Changes in occupancies were also noted when the *Olex2* solvent mask (Dolomanov *et al.*, 2009 ▶) was applied. In contrast, when *PLATON/SQUEEZE* was tested on (2), the occupancies change but not as drastically [guest 1: 81 (1)% to 78 (1)%, guest 2: 45 (1)% to 46 (1)%, solvent 1: 52 (1)% to 51 (1)%, solvent 2: 18 (1)% to 22 (1)%]. Overall, if remedying solvent-accessible void electron density is desired, then it is recommended to fix the free-refined occupancy immediately before removing the electron density. The underlying cause for the changes in site occupancy is currently unclear.

**Figure 4:**
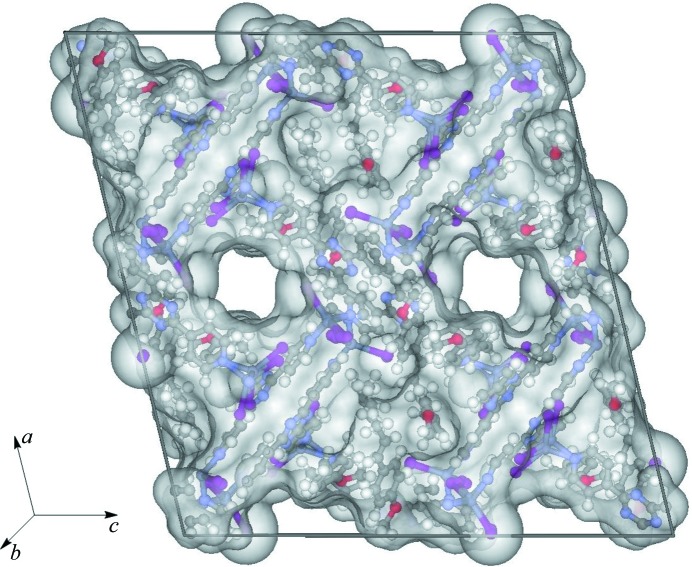
Solvent-accessible voids within the unit cell for (1).

The crystal sponges are three-dimensional polymeric networks that contain infinite networks of solvent-accessible channels (Fig. 4 ▶). Guests and solvent that are within the vdW radius of the host framework can undergo positional stabilization, contributing to their successful identification and refinement. Beyond this region are solvent-accessible voids which accommodate additional guest and solvent molecules that are heavily disordered due to a lack of positional stabilization through sufficient intermolecular interactions. We were initially interested in performing solvent-accessible void electron counting as done by Buchwald and co-workers for their crystal sponge system (Vinogradova *et al.*, 2014 ▶) using the *PLATON/SQUEEZE* program (Spek, 2009 ▶). Two of the main requirements for this procedure are that the data set is essentially complete (especially in regard to the low-angle reflections), and that no large *F*_obs_ − *F*_calc_ (systematic) differences should exist (Spek, 2006 ▶). There are large *F*_obs_ − *F*_calc_ differences present in the current data as indicated through *checkCIF* level C PLAT918 alerts, which are due to pixel saturation arising from the use of high-flux radiation. While this would increase the *wR*_2_ values [*e.g.*, 26% for (1) that triggers a level C *checkCIF* alert since it is larger than the 25% cutoff], from a routine chemical crystallographic standpoint it does not affect the results at hand for the purposes of the current study, especially with satisfactory refinement statistics. However, the use of the *PLATON/SQUEEZE* electron-counting feature is precluded, and removing the problematic reflections would sacrifice the requirement of data completeness, especially within the low-angle shells. If electron-counting analysis is desired, then it is advised that a high completeness is achieved and pixel saturation is avoided. This can be potentially realized through a meticulous data-collection strategy and the careful use of attenuation filters.

Given that there will be significant amounts of unmodeled residual electron density within the solvent-accessible voids, one may consider the use of squeezing/masking techniques in order to lower the magnitude of the maximum electron-density peak and improve the *R*_1_ and *wR*_2_ refinement statistics for publication. It should be noted that *PLATON/SQUEEZE* or the *Olex2* solvent mask were *not* used for (1), (2), (3) or (4). The data did not necessitate their use, and the current refinement statistics pass *checkCIF* tests with the exception of a minor level C alert pertaining to the slightly larger *wR*_2_ value for (1) (26% *versus* the 25% cutoff) and (4) (34%) with the latter arising from high levels of solvent disorder (use of *PLATON/SQUEEZE* did not remedy this level C alert). In general for the crystal sponge systems, the use of procedures to remove residual electron density is justifiable since it belongs to severely disordered solvent and guest molecules through position and occupation that cannot be reasonably modeled; however, the use of these procedures must be done with care and only when warranted. Furthermore, all reasonable attempts should be made to model residual density, and one should verify that it does not originate from incorrect space-group assignment or twinning. Leaving the residual density untreated depicts a more accurate chemical representation of the system and it is suggested that the use of void electron-density squeezing/masking programs should be avoided if the *R*_1_ and *wR*_2_ refinement statistics are reasonable and pass validation tests. Furthermore, disordered density close to the host framework may not be removed since *PLATON/SQUEEZE* maintains a buffer equivalent to rolling a sphere with a 1.2 Å radius over the vdW surface of all atoms, and therefore the benefits of using *PLATON/SQUEEZE* or other solvent-masking programs may be minimal. However, an exception to this is if these programs are able to improve the structural quality of the remaining guests. Then their use is recommended as long as the data are of sufficient quality.

The general conditions needed for the use of *PLATON/SQUEEZE* are (i) acceptable data resolution (0.84 Å), (ii) the remainder of the structure is completed with H atoms in order to generate the vdW surface, (iii) the residual electron density to be squeezed is sufficiently beyond the vdW surface of the modeled structure, and (iv) the treated area is less than approximately 30% of the unit-cell volume (Spek, 2006 ▶). The last requirement is conceivably flexible for three-dimensional polymeric networks such as the crystal sponges, since they inherently contain solvent-accessible voids that will have severely disordered guest/solvent molecules and a larger volume may need to be treated. However, the other requirements should be strictly followed, and either careful in-house data collection or the use of high-flux synchrotron radiation should assist in meeting the resolution requirement. Unlike a previous literature example for a derivative ZnMOF system (Ikemoto *et al.*, 2014 ▶), charges must be balanced before use of these programs, otherwise the structure will have an overall net unbalanced charge which lacks physical meaning. The use of these programs must be documented within the experimental details and CIF, and pretreated data must also be furnished. Whether treatment of residual density is performed or not, a level A alert regarding the presence of large or very large solvent-accessible voids will be triggered and is tolerable. Through the use of synchrotron radiation, (1), (2), (3) and (4) are the first reported crystal sponge systems not requiring the use of *PLATON/SQUEEZE* or other solvent-masking programs to treat residual density within the solvent-accessible voids.

### 2.4. Space-group transformation   

**Figure 5:**
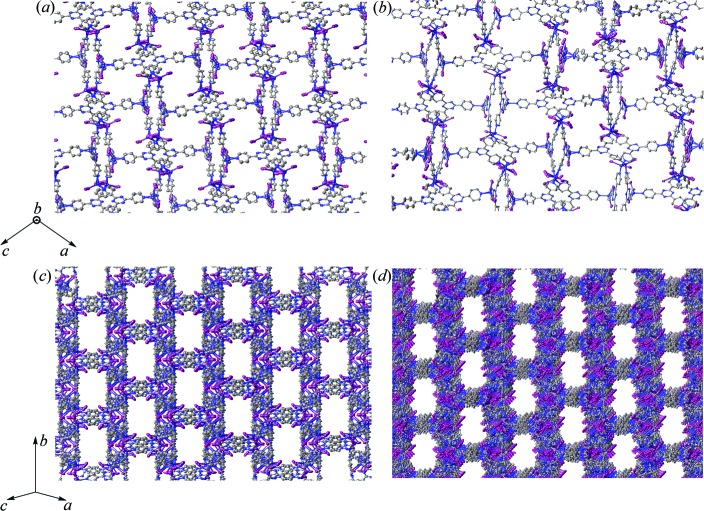
Views of the pores within the host framework for (4) (*a*), (*c*) and (3) (*b*), (*d*). Images (*a*) and (*b*) are viewed along the direction of the *b* axis, whereas images (*c*) and (*d*) are viewed at a 45° angle bisecting the *ac* plane. Hydrogen atoms are hidden for clarity.

The ZnMOF crystal sponges contain a myriad of pores in which the guests can enter and become encapsulated. These pores can be best seen in the direction along and perpendicular to the *b* axis at a 45° angle that bisects the *ac* plane (Fig. 5 ▶). Furthermore, the inclusion of guest molecules in the ZnMOF crystal sponge systems can induce a single-crystal-to-single-crystal transformation. This is reflected by a change in unit-cell dimensions and more markedly through a change in space group (Zhang *et al.*, 2014 ▶). While the inclusion of *trans*-anethole and guaiazulene did induce very minor changes in the unit-cell lengths and β angle relative to (4), the space group did not change from monoclinic *C*2/*c*. As expected, the host frameworks are largely similar and minor distortions in unit-cell dimensions would arise from interaction of the guest with the host.

In contrast, a single-crystal-to-single-crystal transformation was observed for the inclusion of (1*R*)-(−)-menthyl acetate where the space group of (3) changed from *C*2/*c* to *P*2_1_. Furthermore, the *c*-axis length increased to over twice in size [66.990 (6) Å *versus* 31.081 (3) Å]. The Flack *x* parameter was 0.02 (2) and the Hooft *y* parameter (Hooft *et al.*, 2008 ▶) was 0.01 (2), indicating that the absolute stereochemistry of the system has been correctly determined [as a side note, due to limitations of the data-collection strategy at the synchrotron, a Friedel pair coverage of 57% was obtained; however, since (3) contains heavy atoms, the determination of absolute stereochemistry with this level of coverage is reliable]. A closer examination of symmetry elements within the *P*2_1_ space group reveals that it contains twofold screw axes whereas *C*2/*c*, by virtue of *C*-face centering and *c*-glide symmetry (which inherently has an *ac* mirror plane), contains inversion centers, twofold rotation axes and twofold screw axes. Sufficient inclusion of chiral guest into the crystal sponge necessitates the destruction of *c*-glide symmetry because it contains an incompatible improper symmetry operation (inversion center). However, loss of only the inversion centers and an origin shift would afford monoclinic space group *C*_2_. Transformation of *C*2 to *P*2_1_ requires loss of the twofold rotation axes and origin shift, which intrinsically changes the *C*-face-centered lattice to a primitive lattice. The changes in the host pore morphology are more pronounced in (3) compared to the inclusion of guaiazulene and *trans*-ane­thole in the form of minor variations in pore size (Fig. 5 ▶). The small changes in pore morphology, arising through minor changes in the framework torsional angles, necessitates expansion of the unit cell by increasing the *c*-axis length. The other unit-cell axes and β angle do not change significantly between (3) and (4). Thus the minor changes in the host framework, and not just incorporation of (1*R*)-(−)-menthyl acetate alone, likely contribute to the reduction of unit-cell symmetry. Furthermore, the more prevalent disorder of the Zn and I host atoms in (3) could arise from the degree of change in the framework and partial occupancies for 14 (1*R*)-(−)-menthyl acetate guests and one CHCl_3_. In general, (3) contains the largest unit cell compared to other reported crystal sponge examples thus far and contains over 500 non-H atoms. This required significant effort and resources in order to successfully model the structure, which afforded the best Flack *x* parameter for these systems to date.

## 3. Concluding remarks   

Through these studies we were able to greatly improve the simplicity and efficiency of the synthesis of the crystal sponges, obtained and refined data that allowed for the construction of chemically and physically sensible models for guest structure determination, and outlined a complete procedure that encompasses guest inclusion, data collection, processing and refinement. We have outlined problematic cases involving the inclusion of *trans*-anethole and (1*R*)-(−)-menthyl acetate that have been resolved by the use of high-flux synchrotron radiation. Specific to the crystal sponge systems reported to date, (3) contains the largest unit cell with >500 non-H atoms and has the best Flack *x* parameter for absolute structure determination. We have demonstrated that the use of synchrotron radiation is especially helpful for difficult structural cases and for studies requiring a high-throughput workflow necessitating very short data-collection times. These crystallographic procedures will be extremely useful for crystal sponge data collected using in-house diffractometers or with synchrotron sources, and will allow for the construction of reliable models. In order to maintain transparency and accountability, it is vital that the refinement results file and the reflection list/intensities (*hkl* file) are embedded within the CIF for publication, as it will allow other researchers to evaluate data and refinement quality.

Despite the described synthetic and crystallographic guidelines, it is imperative to note that the crystalline sponge method must be used judiciously, and that the results obtained are not always unambiguous or ‘crystal clear’, *per se*. Great care must be taken in interpreting the residual electron density for the guest molecules, especially in cases where the structure is not completely known, or if it exhibits conformational flexibility and thus disorder. With excessive disorder, poor data, over-modeling and/or making erroneous assumptions based upon misguided optimism, the disastrous outcome of drawing incorrect conclusions is very real (Inokuma *et al.*, 2013*b* ▶; Dauter *et al.*, 2014 ▶). It is also important to note that there are limitations to the current ZnMOF crystal sponge system that affect its generality. Not all guests may enter the pores due to their size or their inability to form favorable interactions with the host framework. Compounds that do enter may be severely disordered and may not be discernible. Additionally, the ZnMOF crystal sponge is incompatible with basic amines because of their ability to disrupt the interaction between Zn and the aromatic ligand. Thus it is important for new MOFs to be developed to address these issues. The use of crystal sponges to determine molecular structure and absolute stereochemistry has great promise and, regardless of the MOFs that are developed for this purpose, we believe that the current procedures will continue to be applicable towards obtaining reliable high-quality data.

## 4. Experimental   

### 4.1. Crystal growth and guest inclusion   

The following is a derivative of a previously reported synthetic procedure (Biradha & Fujita, 2002 ▶). To a 12 × 75 mm borosilicate glass test tube charged with tris(4-pyridyl)-1,3,5-triazene (6.3 mg, 0.020 mmol) was added CHCl_3_ (4.2 ml). This mixture was subjected to a few cycles involving brief heating followed by sonication in order to maximize dissolution, and was filtered into another test tube using a Pasteur pipette with a cotton plug. This test tube was placed into a 40 ml scintillation vial and a fresh 0.03 *M* ZnI_2_/MeOH solution (1 ml) was carefully layered on top of the CHCl_3_ solution using a syringe. The interfacial region became opaque. The scintillation vial was sealed and left at ambient temperature for 3 d. The crystals were then gently scraped from the sides of the test tube with a micropipette that had a 1 ml plastic tip which was cut at the end to increase the size of the opening. The crystals were then transferred into another vial with the micropipette and a majority of the solvent was removed. The crystals were washed with CHCl_3_ (4 × 10 ml) by adding and removing solvent with a micropipette to afford very pale yellow crystals with varying morphologies (9.7 mg dry weight, 60% yield). For guest inclusion, multiple crystals were pipetted into a smaller vial and the solvent was carefully removed with a pipette. Enough neat liquid guest to submerge the crystals was immediately added and the crystals were left to soak at room temperature for 2 d. It is important to never let the crystals become dry at any point during synthesis and guest inclusion. The image for Fig. 2 ▶ was captured with a Zeiss Discovery V8 Stereomicroscope using a Plan-Achromat S1.0x Objective, zoom setting of 5.0 and Axiocam MRc color CCD with *Axiovision* software (Carl Zeiss Microscopy, Thornwood, NY).

#### 4.1.1. Crystallographic details   

Crystals were selected in NVH immersion oil and mounted on nylon loops. The loops were placed on a Bruker D8 three-circle fixed chi goniometer with an APEX II CCD detector and 100 K liquid-nitrogen stream generated by an Oxford Cryostream system at the Advanced Photon Source synchrotron in Argonne National Laboratory (ChemMatCARS sector 15 beamline, experimental station ID-B). The data were collected at ∼0.41 Å wavelength using multiple ϕ scans at 0.5° increments with ω offsets. Data processing was performed in the Bruker *APEX2* software suite (Bruker, 2014 ▶), where data integration was done using *SAINT* (Bruker, 2013 ▶) and multi-scan absorption correction was performed using *SADABS* (Bruker, 2012 ▶). The reported data were solved through intrinsic phasing (*SHELXTL XT-2014*) and refined by least-squares refinement on *F* ^2^ (*SHELXL-2014*) (Sheldrick, 2008 ▶). Relevant anomalous dispersion and absorption coefficients (*f*′ and *f*′′) were calculated for the irradiation wavelength used, and all H atoms were added using the riding model. Both Flack *x* and Hooft *y* parameters were calculated using *PLATON/BIJVOETPAIR* (Spek, 2009 ▶) and the validation of (3) was performed using *PLATON/VALIDATION* due to the length of time required to generate a *checkCIF* report (∼15 min).

## Supplementary Material

Crystal structure: contains datablock(s) transanethole_a, guaiazulene_a, menthylacetate2_a, znblank_a. DOI: 10.1107/S2053273314019573/pc5042sup1.cif

Structure factors: contains datablock(s) transanethole_a. DOI: 10.1107/S2053273314019573/pc5042transanethole_asup2.hkl

Structure factors: contains datablock(s) guaiazulene_a. DOI: 10.1107/S2053273314019573/pc5042guaiazulene_asup3.hkl

Structure factors: contains datablock(s) menthylacetate2_a. DOI: 10.1107/S2053273314019573/pc5042menthylacetate2_asup4.hkl

Structure factors: contains datablock(s) znblank_a. DOI: 10.1107/S2053273314019573/pc5042znblank_asup5.hkl

Experimental tables and supplementary SHELXL commands used. DOI: 10.1107/S2053273314019573/pc5042sup6.pdf

CCDC references: 1007929, 1007930, 1007931, 1007932

## Footnotes

1 Supporting information for this paper is available from the IUCr electronic archives (Reference: PC5042).
