# Supplementary material for: Analysis of rapidly synthesized guest-filled porous complexes with synchrotron radiation: practical guidelines for the crystalline sponge method
Source: Acta Crystallogr A Found Adv. 2015 Jan 1;71(Pt 1):46–58. doi: 10.1107/S2053273314019573 (PMC4283468; doi:10.1107/S2053273314019573)
Supplement: Supplementary file 6 [file a-71-00046-sup6.pdf]

Supporting Information

Analysis of Rapidly-Synthesized Guest-Filled Porous Complexes with Synchrotron Radiation: Practical Guidelines for the Crystalline Sponge Method

Timothy R. Ramadhar,[a] Shao-Liang Zheng,[b] Yu-Sheng Chen,[c] Jon Clardy[a]*

[a] *Department of Biological Chemistry and Molecular Pharmacology, Harvard Medical School, 240 Longwood Avenue, Boston, Massachusetts, 02115, United States of America*

[b] *Department of Chemistry and Chemical Biology, Harvard University, 12 Oxford Street, Cambridge, Massachusetts, 02138, United States of America*

[c] *ChemMatCARS, Center for Advanced Radiation Sources, The University of Chicago c/o Advanced Photon Source, Argonne National Laboratory, 9700 South Cass Avenue, Argonne, Illinois, 60439, United States of America*

E-mail: jon_clardy@hms.harvard.edu

Table S1. Experimental details for **1**

	transanethole_a
Crystal data	
Chemical formula	$C_{36}H_{24}I_6N_{12}Zn_3{\cdot}2.44(C_{10}H_{12}O)$
M_r	1943.90
Crystal system, space group	Monoclinic, *C*2/*c*
Temperature (K)	100
a, *b*, *c* (Å)	34.346 (2), 15.1140 (9), 32.5502 (19)
β (°)	103.4150 (13)
V (Å^3)	16436.1 (17)
Z	8
Radiation type	Synchrotron, λ = 0.41317 Å
μ (mm^{-1})	1.68
Crystal size (mm)	0.18 × 0.06 × 0.06
Data collection	
Diffractometer	Bruker D8 goniometer diffractometer
Absorption correction	Multi-scan '*SADABS2012*/1 (Bruker, 2012) was used for absorption correction. wR_2(int) was 0.1148 before and 0.0878 after correction. The Ratio of minimum to maximum transmission is 0.8892. The λ/2 correction factor is 0.0015.'
T_{min}, T_{max}	0.825, 0.928
No. of measured, independent and observed [$I > 2\sigma(I)$] reflections	118509, 13986, 12328
R_{int}	0.065
$(\sin \theta/\lambda)_{max}$ (Å^{-1})	0.596
Refinement	
$R[F^2 > 2\sigma(F^2)]$, $wR(F^2)$, S	0.069, 0.259, 1.04
No. of reflections	13986
No. of parameters	847
No. of restraints	253
H-atom treatment	H-atom parameters constrained
	$w = 1/[\sigma^2(F_o^2) + (0.180P)^2 + 250.P]$ where $P = (F_o^2 + 2F_c^2)/3$
$\Delta\rangle_{max}$, $\Delta\rangle_{min}$ (e Å^{-3})	2.55, -2.50

Computer programs: *APEX2* v2014.3 (Bruker-AXS, 2014), *SAINT* 8.32B (Bruker-AXS, 2013), SHELXTL XT-2014 (Sheldrick, 2008), *SHELXL2014* (Sheldrick, 2008), Olex2 (Dolomanov *et al.*, 2009).

Table S2. Experimental details for **2**

	guaiazulene_a
Crystal data	
Chemical formula	$C_{36}H_{24}I_6N_{12}Zn_3 \cdot 1.26(C_{15}H_{18}) \cdot 0.7(CHCl_3)$
M_r	1915.66
Crystal system, space group	Monoclinic, *C*2/*c*
Temperature (K)	100
a, *b*, *c* (Å)	34.936 (3), 14.9785 (13), 30.825 (3)
β (°)	102.8570 (17)
V (Å^3)	15726 (2)
Z	8
Radiation type	Synchrotron, λ = 0.41321 Å
μ (mm^{-1})	1.78
Crystal size (mm)	0.5 × 0.18 × 0.12
Data collection	
Diffractometer	Bruker D8 goniometer diffractometer
Absorption correction	Multi-scan *SADABS2012*/1 (Bruker, 2012) was used for absorption correction. wR_2(int) was 0.1617 before and 0.0687 after correction. The Ratio of minimum to maximum transmission is 0.7700. The λ/2 correction factor is 0.0015.
T_{min}, T_{max}	0.538, 0.699
No. of measured, independent and observed [$I > 2\sigma(I)$] reflections	91818, 13364, 12388
R_{int}	0.055
$(\sin \theta/\lambda)_{max}$ (Å^{-1})	0.596
Refinement	
$R[F^2 > 2\sigma(F^2)]$, $wR(F^2)$, S	0.085, 0.239, 1.08
No. of reflections	13364
No. of parameters	897
No. of restraints	177
H-atom treatment	H-atom parameters constrained
	$w = 1/[\sigma^2(F_o^2) + (0.080P)^2 + 550.P]$ where $P = (F_o^2 + 2F_c^2)/3$
$\Delta\rangle_{max}$, $\Delta\rangle_{min}$ (e Å^{-3})	2.28, -1.91

Computer programs: *APEX2* v2014.3 (Bruker-AXS, 2014), *SAINT* 8.32B (Bruker-AXS, 2013), SHELXTL XT-2014 (Sheldrick, 2008), *SHELXL2014* (Sheldrick, 2008), Olex2 (Dolomanov *et al.*, 2009).

Table 1. Experimental details for **3**

	menthylacetate2_a
Crystal data	
Chemical formula	$C_{288}H_{192}I_{47.98}N_{96}Zn_{23.99}$·10.21($C_{12}H_{22}O_2$)·0.55($CHCl_3$)
M_r	14743.90
Crystal system, space group	Monoclinic, $P2_1$
Temperature (K)	100
a, b, c (Å)	34.966 (3), 14.8683 (14), 66.990 (6)
β (°)	104.191 (1)
V (Å^3)	33764 (5)
Z	2
Radiation type	Synchrotron, λ = 0.41347 Å
μ (mm^{-1})	1.63
Crystal size (mm)	0.12 × 0.04 × 0.04
Data collection	
Diffractometer	Bruker D8 goniometer diffractometer
Absorption correction	Multi-scan<br>*SADABS2012*/1 (Bruker, 2012) was used for absorption correction. wR_2(int) was 0.0896 before and 0.0769 after correction. The Ratio of minimum to maximum transmission is 0.8759. The λ/2 correction factor is 0.0015.
T_{min}, T_{max}	0.830, 0.948
No. of measured, independent and observed [$I > 2\sigma(I)$] reflections	300285, 95898, 75484
R_{int}	0.081
$(\sin \theta/\lambda)_{max}$ (Å^{-1})	0.596
Refinement	
$R[F^2 > 2\sigma(F^2)]$, $wR(F^2)$, S	0.062, 0.191, 1.02
No. of reflections	95898
No. of parameters	6312
No. of restraints	1137
H-atom treatment	H-atom parameters constrained
	$w = 1/[\sigma^2(F_o^2) + (0.1097P)^2 + 53.2554P]$ where $P = (F_o^2 + 2F_c^2)/3$
$\Delta\rangle_{max}$, $\Delta\rangle_{min}$ (e Å^{-3})	1.48, -1.04
Absolute structure	Flack x determined using 22730 quotients [(I+)-(I-)]/[(I+)+(I-)] (Parsons, S., Flack, H. D. & Wagner, T. (2013). *Acta Cryst.* B**69,** 249-259).
Absolute structure parameter	0.02 (2)

Computer programs: *APEX2* v2014.3 (Bruker-AXS, 2014), *SAINT* 8.32B (Bruker-AXS, 2013), SHELXTL XT-2014 (Sheldrick, 2008), *SHELXL2014* (Sheldrick, 2008), Olex2 (Dolomanov *et al.*, 2009).

Table S4. Experimental details for **4**

	znblank_a
Crystal data	
Chemical formula	$C_{36}H_{24}I_6N_{12}Zn_3 \cdot 1.44(CHCl_3)$
M_r	1754.12
Crystal system, space group	Monoclinic, *C*2/*c*
Temperature (K)	100
a, *b*, *c* (Å)	34.655 (3), 14.7307 (14), 31.081 (3)
β (°)	101.031 (2)
V (Å^3)	15574 (3)
Z	8
Radiation type	Synchrotron, λ = 0.41333 Å
μ (mm^{-1})	1.83
Crystal size (mm)	0.29 × 0.07 × 0.04
Data collection	
Diffractometer	Bruker D8 goniometer diffractometer
Absorption correction	Multi-scan *SADABS2012*/1 (Bruker,2012) was used for absorption correction. wR2(int) was 0.1419 before and 0.0669 after correction. The Ratio of minimum to maximum transmission is 0.8112. The λ/2 correction factor is 0.0015.
T_{min}, T_{max}	0.603, 0.744
No. of measured, independent and observed [$I > 2\sigma(I)$] reflections	115077, 13521, 10586
R_{int}	0.054
(sin θ/λ)$_{max}$ (Å^{-1})	0.596
Refinement	
$R[F^2 > 2\sigma(F^2)]$, $wR(F^2)$, S	0.094, 0.338, 1.16
No. of reflections	13521
No. of parameters	654
No. of restraints	92
H-atom treatment	H-atom parameters constrained
	$w = 1/[\sigma^2(F_o^2) + (0.180P)^2 + 400.P]$ where $P = (F_o^2 + 2F_c^2)/3$
$\Delta\rangle_{max}$, $\Delta\rangle_{min}$ (e Å^{-3})	3.38, -2.88

Computer programs: *APEX2* v2014.3 (Bruker-AXS, 2014), *SAINT* 8.32B (Bruker-AXS, 2013), SHELXTL XT-2014 (Sheldrick, 2008), *SHELXL2014* (Sheldrick, 2008), Olex2 (Dolomanov *et al.*, 2009).

Miscellaneous SHELXL-2014 commands used:

- Change AFIX 137 to AFIX 33 in order to prevent specific methyl H-atoms from rotating in the refinement if they are causing max shift / e.s.d. errors

- Use FREE to remove spurious bonds within the connectivity lists
